# Separation of Dimethyl Sulfide from Wort by Multi-Layer Centrifugal Film Method

**DOI:** 10.3390/foods11182901

**Published:** 2022-09-18

**Authors:** Xiaoyong Dai, Pengyu Wang, Wei Wu, Haoyu Wang, Qing Xu, Zhanyong Li

**Affiliations:** 1 Tianjin Key Laboratory of Integrated Design and On-Line Monitoring for Light Industry & Food Machinery and Equipment, College of Mechanical Engineering, Tianjin University of Science and Technology, Tianjin 300222, China; 2 Tianjin International Joint Research and Development Center of Low-Carbon Green Process Equipment, Tianjin 300222, China; 3 Guangdong Intelligent Filling Technology Limited Company, Foshan 528000, China

**Keywords:** wort boiling, *DMS* removal rate, centrifugal film, low thermal stress, TBA

## Abstract

Installing a separation device for undesirable volatile substances represented by dimethyl sulfide (DMS) in wort boiling systems is a common way to reduce the thermal stress and maintain the beer’s flavor stability (characterized by the thiobarbituric acid (TBA) value), but most of these separation devices need to provide additional vacuum or primary thermal energy. This research shows that it can produce self-evaporation that consumes its own sensible heat when wort is in the state of turbulent film. Therefore, a new gas-liquid separation system named the multilayer centrifugal film-forming device (similar to the spinning cone column (SCC)) is proposed, which can strengthen self-evaporation through wort turbulent film and create gas phase conditions for the separation of undesirable volatile substances. The results show that up to 91.6% of the content of *DMS* in wort could be significantly removed by centrifugal film self-evaporation. The TBA value of wort was reduced by more than 15%, and the wort was not found to be oxidized. Compared with the traditional boiling method, the multi-layer centrifugal film-forming device can significantly save primary energy consumption and reduce energy consumption by 216.4 kJ per liter of wort during the boiling and cooling process.

## 1. Introduction

Dimethyl sulfide (*DMS*) widely exists in nature, which is the most important volatile sulfide [1,2,3]. *DMS* is also a permitted edible flavor according to GB 2760-2014 National Food Safety Standard for Uses of Food Additives. A moderate *DMS* content is beneficial to beer flavor, but excessive *DMS* brings undesirable flavor to beer, such as “raw wine taste” and “boiled corn flavor” [4]. In addition, *DMS* has slight toxicity, which can cause headache, nausea, and vomiting when the *DMS* concentration is low. It may paralyze the central nervous system at a high concentration of *DMS* [5]. The threshold of *DMS* in water is 30 μg/L, but the threshold in beer varies with different types. It is generally believed that the reasonable threshold range is 30–60 μg/L. The concentration of *DMS* in wort should not exceed 100 μg/L, but the target is less than 50 μg/L for some brewers [5,6,7,8,9].

During beer brewing, wort boiling is an energy-intensive process, which consumes about 0.24–0.54 MJ per liter of wort, accounting for 60% of the total steam consumption required by the brewery [6,10]. The main reason for such energy consumption in the boiling stage is the strong boiling carried out to remove the undesirable volatile substances represented by *DMS*. Therefore, the energy recovery system, aimed at recovering the waste heat of boiling secondary steam, has been widely used commercially [11,12]. The heat recovery system seems to have perfectly solved the problem of energy consumption, but the high thermal stress due to strong boiling increases the content of carbonyl compounds, which results in an instability risk regarding the beer flavor [13,14].

Reducing the thermal stress of the wort boiling process by reducing the total boiling evaporation rate has become the research focus of modern boiling technology, which is helpful to reduce the content of carbonyl compounds related to aging in wort and improve the instability of beer flavor. However, it is not conducive to reducing the undesirable volatile substances represented by dimethyl sulfide (*DMS*) in wort below the sensory threshold when the total evaporation rate is too low [11,13]. For example, the low thermal stress boiling technology presented by Kaspar Schulz company keeps the wort temperature at 97.5 °C lower than the boiling point during the boiling stage and strengthens the volatilization of free *DMS* by continuously stirring, which results in an evaporation rate of less than 1%. After boiling, the content of free *DMS* in wort was still as high as 348 μg/L [11]. Scheuren et al. [6] found that S-methyl methionine (SMM) can continue to decompose into *DMS* at a temperature higher than 70 °C while the temperature of wort in whirlpool rest is higher than 70 °C and the evaporation rate at this stage is very low, so it does not have the conditions required for undesirable volatile substances to enter the gas phase. Moreover, Feilner et al. [15] also found that the *DMS* content increased by 124 μg/L when he studied the changes in the *DMS* content in wort before and after whirlpool rest in a medium-sized brewery in Germany. Therefore, the installation of a high-efficiency gas-liquid separation device between the whirlpool tun and wort cooler to remove excess *DMS* in wort has become the research focus of modern low thermal stress boiling technology.

Scheuren et al. [9] put 62 °C wort containing *DMS* into a falling film evaporator heated by steam and guaranteed the wort boiling point was 62 °C by adjusting the pressure. *DMS* volatilized violently and the removal effect of *DMS* improved with the increase in the film flow rate. A boiling system called Merlin developed by Steinecker company includes a conical heating surface [16]. The content of *DMS* in wort reduced from 146 to 49 μg/L when the hot wort was pumped to the top of the conical heating surface and flows down along the conical surface in a film state after whirlpool rest. Another way to separate *DMS* is to inject steam from the bottom of the column and pump wort into the top of the column. The content of *DMS* in steam increases gradually from bottom to top and the undesirable volatile substances in wort are separated. For example, Seldeslachts et al. [16] successfully reduced the *DMS* content in wort from 190 to 20–30 μg/L by adding primary steam to the packed column. Moreover, Dillenburger et al. [10] enhanced the separation of *DMS* in wort through sufficient mass transfer between the secondary steam generated by the kettle tun and wort on the tray, which reduced the total evaporation rate of boiling to 2.7%. These systems need to provide additional thermal energy when separating *DMS*. In addition to increasing primary energy consumption, it also leads to further reaction of wort due to heating.

In addition to gas-liquid separation devices that require additional heat energy, vacuum stripping systems have also been widely studied and used. The common practice is to pump wort with an excessive *DMS* content into a vacuum vessel and the large amount of steam brought by vacuum stripping creates gas phase conditions for the effective separation of *DMS*. Kaspar Schulz company adopts a similar method, which boils wort at a holding temperature of 97.5 °C (total evaporation rate < 1%). After whirlpool rest, the wort is vacuumized to a gauge pressure of —0.8 bar in the film state. At this time, the total evaporation rate of wort is about 7%, and the *DMS* content in wort is successfully reduced from 348 to 58 μg/L [11]. A wort film forms on the inner wall when the wort enters from the tangent inlet of the vacuum vessel. Ziemann company and NERB company have developed efficient *DMS* separation devices based on this principle. Ziemann company separates excess *DMS* in wort by vacuum film stripping after whirlpool rest, which achieves the purpose of shortening the boiling time [11]. NERB company uses its film stripping system to separate the *DMS* in superheated wort flowing through the external heater during the boiling process and it is further strengthened by vacuum film stripping, which can make the content of *DMS* in wort lower than 35 μg/L [11]. Vacuum stripping is similar to equilibrium distillation and its effect is worse than atmospheric evaporation (simple distillation) under the same evaporation capacity. In addition, generating a vacuum requires additional energy and water, and the system must be strong enough to withstand the vacuum pressure [17].

Feilner et al. [15,17,18,19] developed a gas-liquid separation system of self-evaporation using the sensible heat of wort in the state of turbulent film. The system is installed between the whirlpool tun and the wort cooler to separate the excessive undesirable volatile substances in the wort after whirlpool rest. For this new system, the wort rotates and accelerates in a nozzle on the top of the tank, and then forms a turbulent film on the inner wall of the tank. The gas is introduced from the bottom of the tank to improve the concentration gradient of undesirable volatile substances in the gas–liquid boundary layer on the surface of wort turbulent film to strengthen the separation of undesirable volatile substances. The steam generated by self-evaporation and the incoming gas are discharged from the top exhaust port. Experiments show that the *DMS* removal rate can reach 70%. This system solves the problem of additional energy or vacuum being required when strengthening the separation of *DMS*. However, the film thickness, turbulence degree, and the efficiency of additional gas can still be further improved to enhance mass transfer.

Spinning cone columns (SCCs) have been widely used in the brewing field. For example, it was found that SCCs can not only separate alcohol in beer effectively but also separate other flavor compounds well when Catarino and Mendes used SCC to remove alcohol in beer to produce alcohol-free beer [20]. Belisario Sanchez proved that SCC distillation is a dealcoholizing technology with the least damage to wine phenolic compounds through an experiment in alcohol was removed from 19 kinds of wine [21,22]. The research of Huerta-Pérez and Pérez-Correa shows that SCC can improve the yield of wine and beer dealcoholizing products [23]. SCC is also a good film-forming device. Makarytchev et al. [24] carried out dry column computational fluid dynamics (CFD) simulation on SCC, which showed that unstable gas flow with pressure pulsation was generated when the Reynolds number was greater than 100. Moreover, the mass transfer and pressure drop of SCC under different gas-liquid flow rates were also further studied [25]. A recent simulation by Bae et al. [26] presented that in SCC, distinct internal flows were caused, and the average velocity magnitude was enlarged by about 2.5 times while the mass transfer area was estimated to increase by between 20% and 40% due to the generated liquid droplets.

SCC is an excellent centrifugal film-forming device and an efficient volatile component separation system, which shows that it is a potential device for the removal of *DMS* by self-sensible heat of wort in the state of turbulent film. Therefore, this paper aimed to develop a wort self-evaporation system by boiling wort at 98 °C as the research object [27]. Its film-forming principle is similar to SCC. The purpose was to reduce the primary energy consumption and thermal stress during the process of wort boiling. Wort is easily oxidized in the turbulent film state. Therefore, during this experiment, not only were the *DMS* content, self-evaporation rate, and TBA value of key undesirable volatile substances measured but the chromaticity of wort was also measured and compared with the wort before separation to evaluate the performance of the separation device.

## 2. Materials and Methods

### 2.1. Materials and Reagents

Pearson malt was purchased from Weyermann, Germany. Hops were purchased from Perle, Germany. The test reagents used in the experiment are shown in Table 1.

### 2.2. Experimental Rig and Operation Parameters

**Figure 1:**
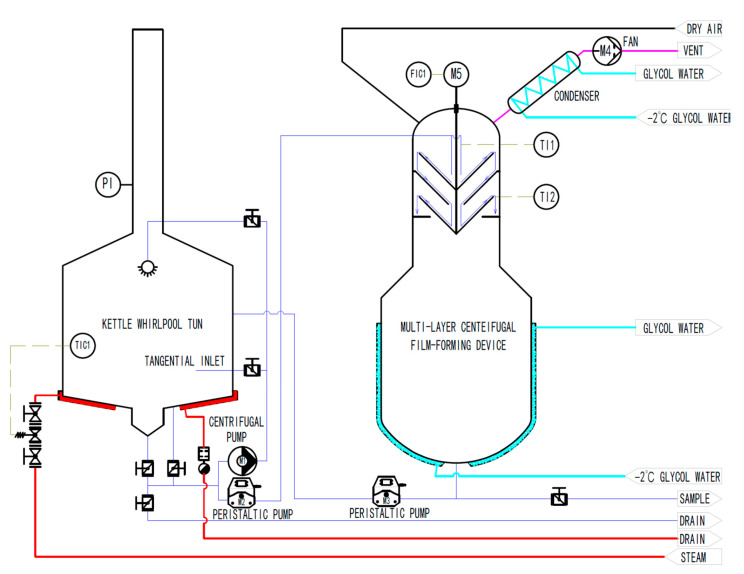
Piping and instrumentation diagram.

**Figure 2:**
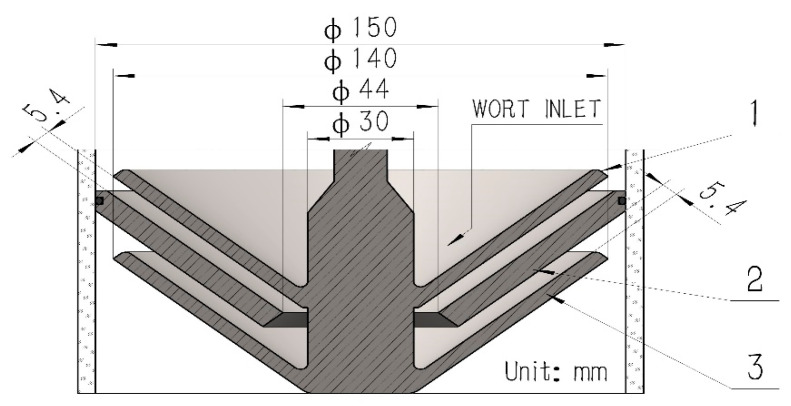
Detailed drawing of the multilayer centrifugal film-forming device. 1-Rotating cone; 2-Fixed cone; 3-Rotating cone.

The piping and instrumentation diagram of the system is shown in Figure 1, which is composed of a self-made traditional kettle whirlpool tun, multi-layer wort centrifugal film-forming device (as shown in Figure 2), pump, pipeline, valves, and various sensors. The net volume of the kettle is 20 L, with a steam jacket at the bottom, and the temperature of wort is controlled by the temperature sensor TIC1 and the steam solenoid valve. The temperature control accuracy is ±0.2 °C. The centrifugal pump M1 is used to pump the wort from the bottom of the kettle into the tangential inlet to separate the hot trub in the wort. The initial wort in the kettle can be pumped into the top rotating cone of the multilayer centrifugal film-forming device through the peristaltic pump M2. The multilayer centrifugal film-forming device is driven by a motor M5 with a power of 60 W and its rotation speed is controlled by the frequency converter FIC1. *K*-type thermocouples TI1 and TI2 are, respectively, used to measure the temperature of wort entering and leaving the multilayer centrifugal film-forming device. The secondary steam generated by the self-evaporation of wort is sucked into the condenser installed on the top of the multilayer centrifugal film-forming device by the fan for condensation and collected for analysis. In order to avoid the vacuum caused by the fan, dry air must be introduced when the fan is started to ensure the atmospheric pressure in the multilayer centrifugal film-forming device. After the wort flows through the multilayer centrifugal film-forming device, it falls into a 10 L buffer tank with a glycol cooling jacket for rapid cooling. The cooled wort is sampled through the sampling port installed at the bottom of the buffer tank for detection.

The wort production in the experiments started with milling the malt and mixing 5 kg of milled malt with 20 L of 72 °C hot water to obtain a mash with a temperature lower than 64 °C. Then, the mash was heated up to 64 °C and maintained at this temperature for 90 min. Owing to enzymatic conversion processes, this malt particle suspension turned into a sugary solution: the wort. After separation of the insoluble malt components during lautering and sparging (with 80 °C hot water), 20 L of wort with a specific gravity of 1.040 was collected. The collected wort was then heated up to 98 °C and boiled with low thermal stress at the holding temperature of 98 °C for 60 min, with a total evaporation rate of less than 1% in the kettle whirlpool tun. The wort production ended with the hot trub separation, during which the centrifugal pump M1 and tangent inlet valve were opened for 10 min of circulation, and then the pump and valve were closed for 20 min of whirlpool rest. After the whirlpool rest, the hot wort was pumped into the centrifugal film-forming device by the peristaltic pump M2 and separated at different speeds. The separated wort was sampled and filled into headspace bottles. Then, the headspace bottles were placed in a low-temperature ice water bath (DFY-5L/20, Gongyi Yuhua Instrument Co., Ltd., Gongyi, China) for rapid cooling.

### 2.3. Analytical Methods

Determination of the *DMS* content was used to analyze the performance of the centrifugal film-forming device. Then, 20 mL headspace bottles with 10 mL of cold wort were placed in a 50 °C electrothermal constant temperature water bath for 30 min and measured based on ASBC (American Association of Brewing Chemists) analysis methods [28] (pp. 91–95) by a gas chromatograph (Nexis GC-2030, Shimadzu Co., Ltd., Kyoto, Japan). During the measurement, helium gas was used as the carrier at a flow rate of 14 mL/min with a diversion ratio of 10. The column (HP-INNOWAX size at 30 m × 0.250 mm × 0.25 μm, Agilent Technologies, Inc., Santa Clara, CA, USA) was heated to 40 °C and held for 5 min, then heated to 120 °C at a heating rate of 5 °C/min, and heated continuously to 200 °C at a heating rate of 10 °C/min, and finally maintained for 1 min.

In order to prove the positive effect of the 98 °C holding temperature boiling method combined with the multi-layer centrifugal film-forming device on reducing the TBA value of wort, 10 mL cold wort samples were centrifuged with a centrifuge (H1650, Hunan, China) at 10,000 r/min until they were clarified. After that, 5 mL of the top supernatant was mixed with 2 mL of TBA solution with a mass concentration of 3.3 g/L. The solvent was 50% acetic acid solution. After mixing, the mixture was kept in a water bath at 60 °C for 60 min and then cooled down quickly. An ultraviolet-visible spectrophotometer (Type 752, Shanghai, China) was used for colorimetry at 530 nm and a pure water sample was selected as the blank sample for calibration. The TBA value was defined as the absorbance value [29,30].

Color analysis of wort can indicate whether wort is oxidized. The wort color was measured based on the ASBC analysis methods [28] (pp. 188–189, pp. 237–238). The cold wort sample was filtered with a 0.45μm hydrophilic polytetrafluoroethylene disc filter (the first 2 mL of wort was discarded). An ultraviolet visible spectrophotometer (Type 752, Shanghai) was used for colorimetry at 430 nm and a pure water sample was selected as the blank sample for calibration. The absorbance value of A430 was detected and Chromaticity F was calculated by 25 multiplied by A430.

All the above experiments were repeated at least three times.

### 2.4. Wort Self-Evaporation Rate E

It was found that wort undergoes self-evaporation in the state of centrifugal turbulent film, consuming its own sensible heat. The secondary steam produced by self-evaporation provides an effective condition for vapor-liquid separation of *DMS*.

To evaluate the performance of the multi-layer centrifugal film device, the wort self-evaporation ratio *E*, *DMS* removal ratio $E_{DMS}$, and *DMS* loss rate φ were defined by the following equations:

(1) $$E=\frac{V_{s}}{V_{i}}\times 100\%$$

(2) $$E_{DMS}=1-\frac{\left(V_{i}-V_{s}\right)\times C_{o}}{V_{i}\times C_{i}}\times 100\%\;$$

(3) $$\varphi =1-\frac{V_{s}\times C_{s}}{V_{i}\times C_{i}-\left(V_{i}-V_{s}\right)\times C_{o}}\times 100\%\;$$

where *V_s_* represents the volumetric flow rate of condensate; *V_i_* is the volumetric flow rate of feeding wort, L/h; $C_{o}$ is the concentration of *DMS* in the outlet wort, μg/L; and $C_{i}$ is the concentration of *DMS* in feeding wort, μg/L.

### 2.5. Analysis of Self-Evaporation Energy Balance

In order to analyze the energy balance of wort self-evaporation, *K*-type thermocouples (TI1 and TI2) were used to measure the inlet and outlet temperature of wort, respectively. The calculation formulas of the latent heat required for steam evaporation (*E_L_*), sensible heat released from wort (*E_S_*), and self-evaporation thermal efficiency (*γ*) are shown as follows:

(4) $$E_{L}=V_{s}\times \rho_{S}\times H$$

(5) $$E_{S}=\left(V_{i}-V_{s}\right)\times \rho_{i}\times Cp\times \left(T_{i}-T_{o}\right)$$

(6) $$\gamma =\frac{E_{L}}{E_{s}}\times 100\%$$

where *H* is the latent heat of secondary steam, kJ/kg; *Cp* is the specific heat capacity of wort, kJ/(kg·°C); $\rho_{i}$ is the density of wort, kg/m^3^; $\rho_{s}$ is the density of condensate, kg/m^3^; and $T_{i}$ and $T_{o}$ refer to the inlet and outlet temperature of wort, respectively, °C. In order to simplify the calculation, the latent heat of secondary steam was taken as the latent heat of pure water, which is about 2258 kJ/kg, and its density was equal to 1000 kg/m^3^. The wort density was 1040 kg/m^3^ and the sensible heat of wort was 4.1 kJ/(kg·°C).

## 3. Results and Discussion

### 3.1. Removal Effect of DMS in Wort

The *DMS* content of the wort boiled at the holding temperature of 98 °C for 60 min was 630 μg/L. The performance of the multi-layer centrifugal film-forming device was investigated with two wort feeding rates (40 and 80 L/h) and different rotation rates from 200 to 1200 rpm, with an increasing interval of 200 rpm at atmospheric pressure. The temperature of the initial wort was 98 °C. Due to the different feeding flow rates of wort in the transmission pipeline, the energy loss in the transmission process differed slightly. The temperature of wort entering the multi-layer centrifugal film-forming device was 95.9 and 97 °C, respectively, when the wort feeding flow rate was 40 and 80 L/h.

#### 3.1.1. Changes in DMS in Wort

**Figure 3:**
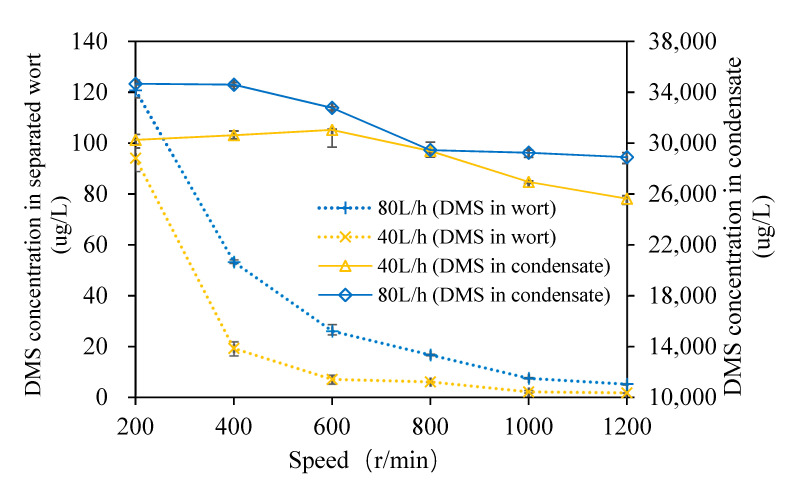
Change in the *DMS* content of wort and condensate.

**Figure 4:**
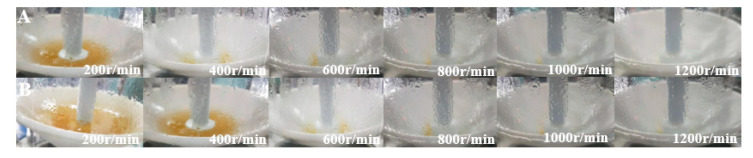
Film forming characteristics of wort at different speeds: (**A**) 40 L/h; (**B**) 80 L/h.

The changes in the *DMS* content of the wort and condensate with the rotation speed of the multilayer centrifugal film-forming device are shown in Figure 3. It can be seen from the figure that when the wort feeding flow was 40 L/h and the rotation speed was from 200 to 1200 rpm with an increasing interval of 200 rpm at atmospheric pressure, the *DMS* content in wort was 94.1, 19.3, 7.1, 6.1, 2.2, and 1.8 μg/L, respectively, which are within the sensory threshold (100 μg/L). The *DMS* content of wort decreased gradually with the increase in the rotating speed. Bae et al. [26] found that there were distinct internal flows in SCC, and the droplets generated at the edge of the rotating cone increased the mass transfer area by 20% to 40% when the rotating speed increased. Feilner et al. [17] also demonstrated that the removal rate of *DMS* is positively correlated with the turbulence degree of wort film. With the increase in the rotating speed, the mass transfer area and the degree of turbulence of wort both increased, which lead to good separation of *DMS*. The *DMS* content in separated wort decreased rapidly when the rotating speed increased from 200 to 400 r/min. This was due to the wort incompletely forming a film at the bottom of the rotating cone, and there was more effusion (as shown in Figure 4A) when the rotating speed was 200 r/min. When the rotating speed increased to 400 r/min, the effect of wort film formation was good, and there was only a small amount of wort effusion at the bottom. When the rotating speed was increased, there was basically no effusion at the bottom of the cone. At this time, the *DMS* content in the separated wort was at a low level, and the content difference in wort was very small.

It can be seen from Figure 3 that the *DMS* content in wort was 120.8 53.3, 26.2, 16.8, 7.6, and 5.3 μg/L, respectively, when the wort feeding flow was 80 L/h and the rotation speed ranged from 200 to 1,200 rpm with an increasing interval of 200 rpm at atmospheric pressure. With the increase in the rotating speed, the *DMS* content in wort decreased gradually, which is consistent with the trend observed when the wort feeding flow was 40 L/h. The *DMS* content in separated wort decreased rapidly when the rotating speed increased from 200 to 600 r/min. When the rotating speed was 200 r/min, the *DMS* content in wort was slightly higher than the sensory threshold (100 μg/L). As can be seen from Figure 4B, there was more wort effusion in the cone bottom, and this meant that the film-forming effect was poor, so the separation effect of *DMS* in wort was poor when the rotating speed was 200 and 400 r/min. When the rotating speeds were the same, the larger the feeding flow, the higher the *DMS* content in wort. This is because the thickness of the wort film on the rotating cone and the fixed cone of the multi-layer centrifugal film-forming device increased with the increase in the feeding flow rate, which led to an increase in the migration resistance of *DMS* in liquid wort. In addition, due to the low inlet temperature when the feeding flow rate was 40 L/h, the relative volatility of the volatile components was slightly higher [17], which also intensified this trend. The above analyses show that the multi-layer centrifugal film-forming device had a good separation effect on *DMS* in wort. When the rotating speed was 400 r/min, the *DMS* content in wort was far lower than the sensory threshold, so it was not significant to continue to increase the rotating speed.

The secondary steam produced by self-evaporation of the wort was condensed, and its *DMS* content was also detected, which is shown in Figure 3. It can be seen from the figure that when the wort feeding flow was 40 L/h and the rotation speed ranged from 200 to 1200 rpm with an increasing interval of 200 rpm at atmospheric pressure, the *DMS* content in the condensate was 30,254, 30,626, 31,044, 29,388, 26,952, and 25,630 μg/L, respectively. When the wort feeding flow rate was 80 L/h and the rotation speed ranged from 200 to 1200 rpm with an increasing interval of 200 rpm at atmospheric pressure, the *DMS* content in the condensate was 34,673, 34,600, 32,777, 29,454, 29,248, and 28,896 μg/L, respectively. It was reported that the content of *DMS* in steam condensate is about 10,000 μg/L when primary steam stripping is used to remove *DMS* [11]. This shows that a large amount of *DMS* is transferred to the condensate, and the multi-layer centrifugal film-forming device can strengthen the separation of *DMS* well. It also demonstrates that the separation effect of centrifugal film self-evaporation on *DMS* in wort is much higher than that of steam stripping. The *DMS* loss rate calculated by Formula 3 ranged from 12.04%~19.51% and 13.32%~27.63% for a feeding flow of 40 and 80 L/h, respectively. This indicates that the *DMS* separated from wort was not completely condensed.

#### 3.1.2. The Change in the DMS Removal Rate in Wort

**Figure 5:**
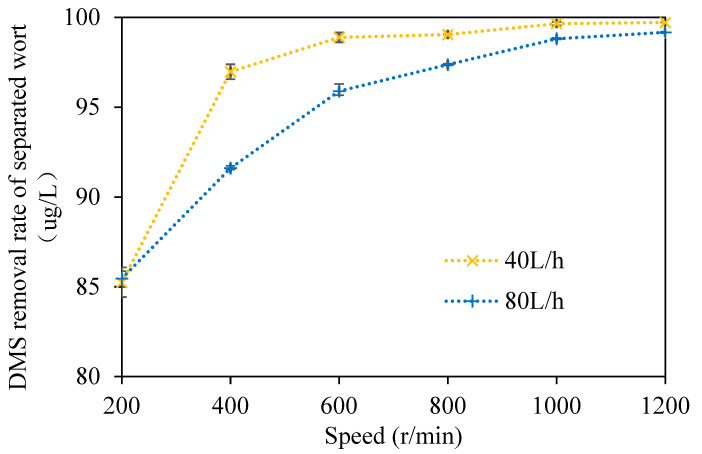
Removal rate of *DMS* in separated wort.

In order to more intuitively show the removal effect of *DMS* in wort using the multi-layer centrifugal film-forming device, the removal rate of *DMS* in wort was studied in this paper, as shown in Figure 5. It can be seen from the figure that when the wort feeding flow was 40 L/h and the rotation speed ranged from 200 to 1200 rpm with an increasing interval of 200 rpm at atmospheric pressure, the removal rates of *DMS* in wort were 85.2%, 97%, 98.9%, 99%, 99.7%, and 99.7%, respectively. With the increase in the rotating speed, the removal rate of *DMS* in separated wort gradually increased. The removal rate of *DMS* in wort was 97% when the rotating speed was 400 r/min, and the change in the *DMS* removal rate in wort was slight when the rotating speed continued to increase. When the rotating speed was 200 r/min, there was a small amount of wort effusion at the bottom of the rotating cone and the wort was incompletely filmed, which led to the low removal rate of *DMS* in wort. When the wort feeding flow rate was 80 L/h and the rotation speed ranged from 200 to 1200 rpm with an increasing interval of 200 rpm at atmospheric pressure, the removal rates of *DMS* in wort were 85.5%, 91.6%, 95.9%, 97.4%, 98.8%, and 99.2%, respectively. With the increase in the rotating speed, the removal rate of *DMS* in separated wort also increased gradually, which is consistent with the trend showing that when the wort feeding flow was 40 L/h, the removal rate of *DMS* in wort was slightly lower at the same rotating speed.

Feilner et al. [15,17,18,19] developed a gas-liquid separation system of self-evaporation using the sensible heat of wort in the state of turbulent film. Under the condition of additional gas enhancement, this system could remove about 70% of *DMS* in wort. Kaspar Schulz successfully reduced the *DMS* content in wort from 348 to 58 μg/L by vacuum film stripping at −0.8 bar (gauge pressure) after whirlpool rest. At this time, the total evaporation rate of wort was 7% and the removal rate was about 83.3% [11]. The removal effect of the multi-layer centrifugal film on *DMS* in wort is better than the above two systems. Moreover, no additional vacuum or gas was required to strengthen it. There was more wort effusion at the bottom of the rotating cone when the rotating speed was 200 r/min and there was still a small amount of wort effusion at the bottom of the rotating cone when the rotating speed was 400 r/min. Therefore, the removal rate of *DMS* in wort was relatively low. The removal rate of *DMS* in wort reached 97.4% when the rotating speed was 800 r/min, and it changed little when the rotating speed continued to increase. The above research shows that the removal efficiency of *DMS* in wort depends on the wort film-forming effect of the multilayer centrifugal film-forming device.

#### 3.1.3. Relationship between the DMS Removal Rate and Self-Evaporation Rate

**Figure 6:**
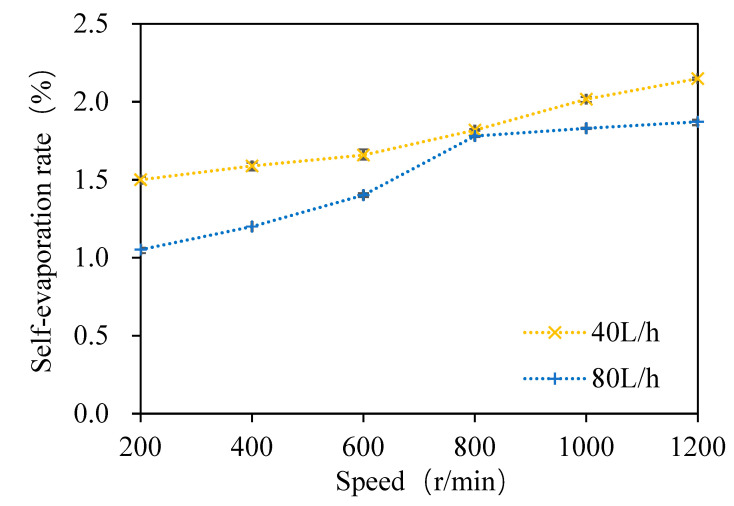
Variation of the self-evaporation rate with the rotating speed.

As shown in Figure 6, when the wort feeding flow was 40 L/h and the rotation speed ranged from 200 to 1200 rpm with an increasing interval of 200 rpm at atmospheric pressure, the self-evaporation rate of wort was 1.5%, 1.6%, 1.7%, 1.8%, 2%, and 2.2%, respectively. With the increase in the rotating speed, the self-evaporation rate of wort increased gradually. Similarly, when the wort feeding flow rate was 80 L/h and the rotation speed ranged from 200 to 1200 rpm with an increasing interval of 200 rpm at atmospheric pressure, the self-evaporation rate of wort was 1.1%, 1.2%, 1.4%, 1.8%, 1.8%, and 1.9%, respectively. With the increase in the rotating speed, the self-evaporation rate of wort increased gradually, but it was slightly lower than that of the wort feeding flow rate of 40 L/h. Under both feeding flow rates, the evaporation rate and removal rate increased with the increase in the rotating speed. In order to further reveal the separation mechanism of *DMS* in wort in the multi-layer centrifugal film-forming device, the relationship between the *DMS* removal rate and the self-evaporation rate in wort with a feeding flow rate of 40 L/h was studied in this paper.

**Figure 7:**
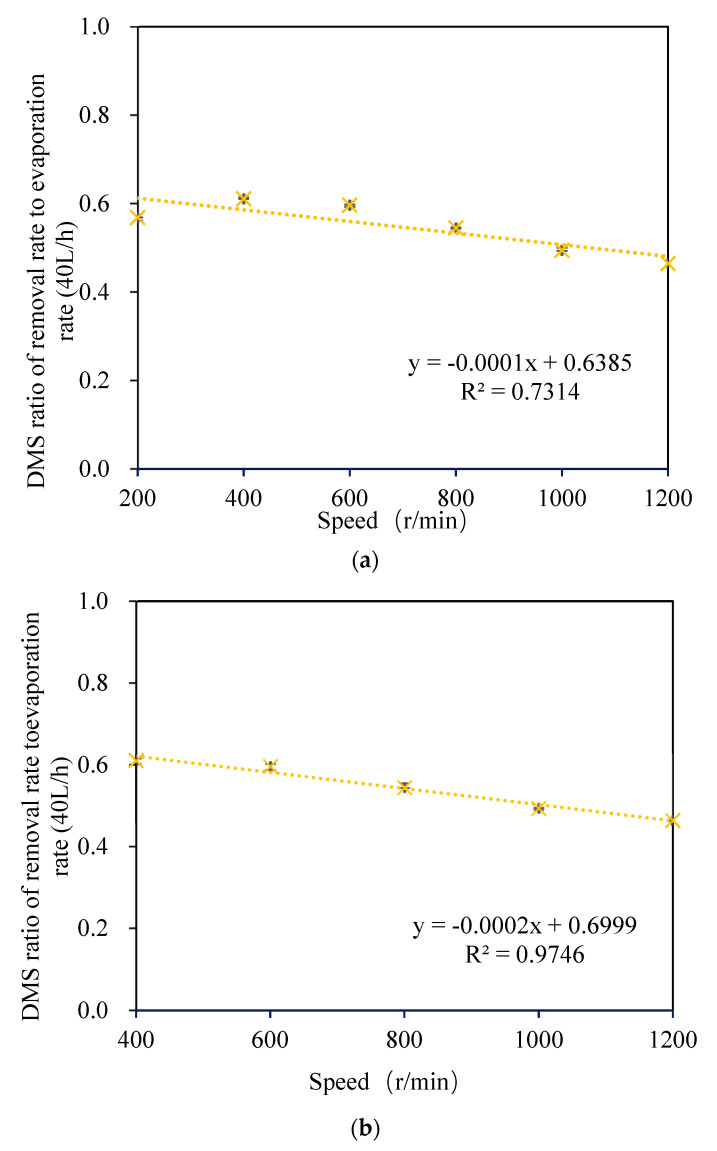
Variation of the ratio of the *DMS* removal rate to the wort self-evaporation rate with the rotating speed: (**a**) 200−1200 r/min; (**b**) 400−1200 r/min.

As shown in Figure 7, by comparing the relationship between the removal ratio of *DMS* to the self-evaporation rate in wort and the rotation speed, no significant linear relationship between the two was observed at a rotation speed of 200–1200 r/min (as shown in Figure 7a). However, a significant linear relationship was observed when the rotating speed was 400–1200 r/min (as shown in Figure 7b). This is because the effect of wort film formation was not ideal when the rotating speed was 200 r/min, and there was more wort effusion at the bottom of the cone (as shown in Figure 4A). However, a significant linear relationship was observed when the rotating speed was 400–1200 r/min (as shown in Figure 7b). It can be concluded that the *DMS* removal rate has a good correlation with the self-evaporation rate if the effect of wort film formation is ideal. This is consistent with the research conclusion of Feilner et al. regarding the wort turbulent film self-evaporation system [17]. When the wort flow rate was 80 L/h and the rotating speed was 200–600 r/min, there was no significant linear relationship between the *DMS* removal rate and self-evaporation rate in wort due to the different degrees of effusion at the cone bottom (as shown in Figure 4B).

### 3.2. Analysis of Self-Evaporation Energy Balance

**Figure 8:**
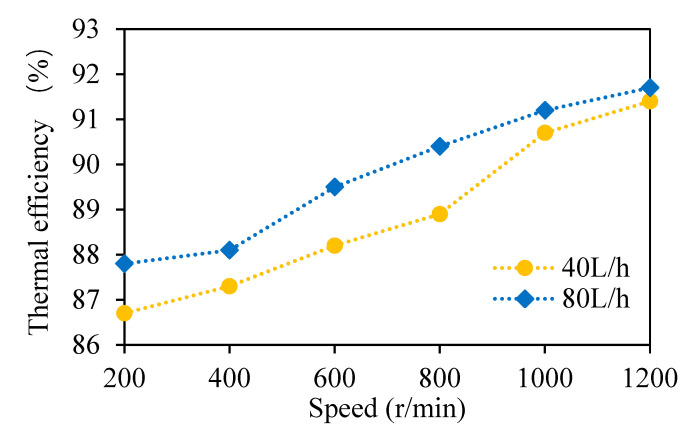
Thermal efficiency of wort self-evaporation.

The temperatures of the wort entering the multi-layer centrifugal film-forming device were 95.9 and 97 °C, respectively, when the wort feeding flow rate was 40 and 80 L/h. The temperatures of the wort leaving the multi-layer centrifugal film-forming device are listed in Table 2. The wort outlet temperatures decreased with the increase in the rotating speed of the multi-layer centrifugal film-forming device. This is because with the increase in the speed, the self-evaporation rate of wort increased, and the wort had to provide more sensible heat, as described in Section 3.1.3. When the feeding flow rate was 40 and 80 L/h, the latent heat required for steam evaporation and the sensible heat released by wort at different speeds were calculated using Formula 4 and Formula 5, respectively. It can be seen from Table 2 that the sensible heat (*E_S_*) released by wort was higher than the latent heat (*E_L_*) required for steam evaporation, and most of the released sensible heat was transformed into the latent heat of steam. In order to show this difference more intuitively, the self-evaporation thermal efficiency was calculated by Formula 6. As shown in Figure 8, the thermal efficiency increased with the increase in the wort feeding flow rate and rotating speed. The maximum thermal efficiency was 91.7%, which means an energy loss of 8.3% at least. This is because the whole system cannot be completely insulated. In conclusion, the self-evaporation of wort provided an effective condition for vapor-liquid separation of *DMS*.

### 3.3. Analysis of Energy Saving Potential

Previous studies have shown that the total evaporation rate of the boiling system is between 1 and 8% [10,15,31]. Although some researchers have studied the removal character of *DMS* by wort self-evaporation, most operations still need additional heat, vacuum, or gas. In this paper, the thermal energy consumed by boiling evaporation was calculated by Equation (7) and is listed in Table 3:

(7) $$E_{W}=E\times V_{W}\times \rho_{i}\times H$$

where *E_W_* is the latent heat of evaporation required per liter of wort, kJ; and *V_W_* is 1 L wort.

The multi-layer centrifugal film-forming device used in this study did not need to consume additional heat or vacuum; only the sensible heat of wort itself was needed. This also reduced the energy consumption of the wort cooling in the following step, and the total boiling evaporation rate could be significantly reduced to 1% through the strengthening of the multi-layer centrifugal film-forming device. When the feeding flow rate was 40 or 80 L/h and the rotating speed was 400 rpm, qualified wort with a *DMS* content far below the threshold could be obtained. Compared with traditional boiling process, 164.4 kJ/L of primary energy consumption could be saved per liter of wort, and 52.0 kJ/L of wort cooling energy consumption could be reduced (flow rate was 40 L/h, and the starting temperature of wort cooling was 97 °C). In addition, the multi-layer centrifugal film-forming device unit could be easily integrated into the existing brewing system.

### 3.4. Variation of TBA Value of Wort

**Figure 9:**
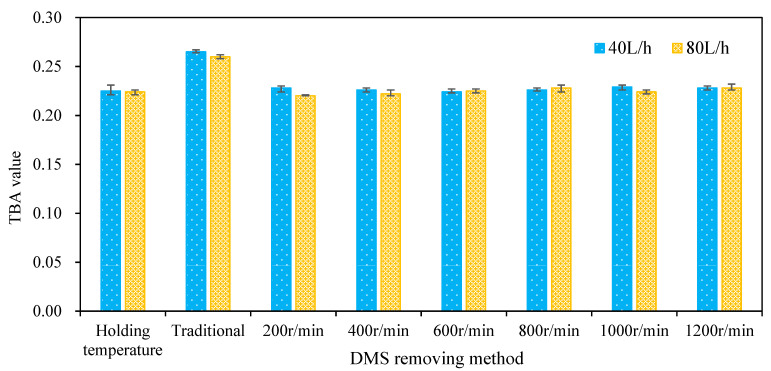
Effect of the *DMS* removal method in wort on the TBA value.

The TBA value is an important parameter used to characterize wort aging [13,14]. Figure 9 shows the TBA value of wort after boiling at a holding temperature of 98 °C and separation by the centrifugal film, which is also compared with traditional boiling. It can be seen from the figure that the corresponding TBA values of wort were 0.225 and 0.224, respectively, when the holding temperature boiling method was adopted and the feeding flow was 40 and 80 L/h while the TBA values were 0.265 and 0.26, respectively, when the traditional boiling method was adopted. The TBA values of separated wort were 0.228, 0.226, 0.224, 0.226, 0.229, and 0.228, respectively, when the wort flow was 40 L/h and the rotation speed ranged from 200 to 1200 rpm with an increasing interval of 200 rpm at atmospheric pressure. The TBA value of separated wort at different rotating speeds was basically the same, which is lower than that obtained by the traditional boiling method. When the wort flow rate was 80 L/h, it also showed the same characteristics. In conclusion, the wort feeding flow and rotation speed have no significant effect on the wort TBA value. Moreover, the TBA value of wort obtained by 98 °C holding temperature boiling combined with multi-layer centrifugal film separation is about 15% lower than that obtained by the traditional boiling method. This shows that the 98 °C holding temperature boiling method combined with the multi-layer centrifugal film-forming device has a positive effect on reducing the TBA value of wort.

### 3.5. Color Change and Oxidation of Wort

**Figure 10:**
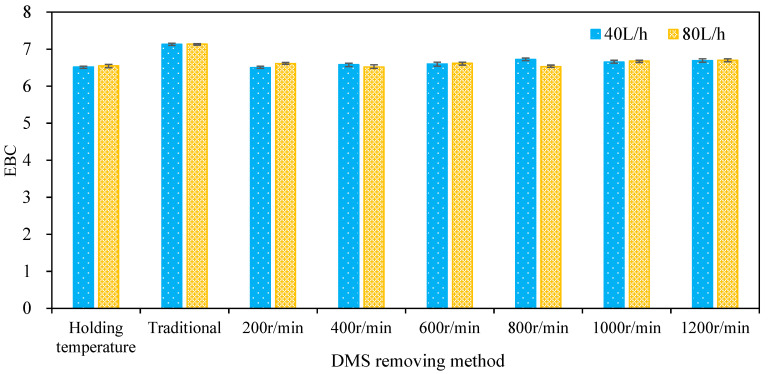
Effect of the *DMS* removal method on wort color.

The separation of undesirable volatile substances by the enhancement of wort turbulent film is often considered to cause oxidation [15]. The color change caused by oxidation is usually unacceptable [15], which is an important index used to evaluate the feasibility of a multi-layer centrifugal film to enhance the self-evaporation of hot wort. In this paper, the color of wort separated by the multi-layer centrifugal film-forming device was detected and evaluated, as shown in Figure 10. It can be seen from the figure that when the wort flow was 40 and 80 L/h, the wort EBC values obtained by holding temperature boiling were 6.52 and 6.55, respectively, and the wort EBC values obtained by traditional boiling were 7.13 and 7.14, respectively. The EBC values of wort were 6.5, 6.58, 6.6, 6.72, 6.65, and 6.69, respectively, when the wort feeding flow was 40 L/h and the rotation speed ranged from 200 to 1200 rpm with an increasing interval of 200 rpm at atmospheric pressure while the EBC values of wort were 6.61, 6.52, 6.62, 6.53, 6.68, and 6.7, respectively, when the wort feeding flow was 80 L/h. The EBC value of wort obtained at different rotating speeds showed little difference, which is close to the EBC value of wort after the holding temperature boiling process, and slightly lower than that obtained by traditional boiling. This shows that the self-evaporation of hot wort enhanced by the multi-layer centrifugal film-forming device does not cause oxidation of the wort. One possible explanation for this is that steam escapes from the wort film continuously to form a phase boundary layer, and there is basically only steam in this phase boundary layer. The air in the chamber of the multi-layer centrifugal film-forming device cannot overcome this steam layer and react with the wort film [15].

## 4. Conclusions

In this paper, a multi-layer centrifugal film-forming device was used to enhance the self-evaporation of hot wort to remove *DMS* from wort. This research shows that the multi-layer centrifugal film-forming device could efficiently remove *DMS* in wort by turbulent film self-evaporation without additional energy and vacuum. Moreover, it has a certain energy-saving potential and did not cause oxidation of the wort, which is helpful for improving the stability of beer flavor by continuously reducing the total wort boiling evaporation rate. The main conclusions are as follows:

(1) The content of *DMS* in wort gradually decreased with the increase in the rotating speed of the multi-layer centrifugal film-forming device. The removal rate of *DMS* in wort reached 91.6% when the rotating speed was 400 r/min, and the content of *DMS* in wort was much lower than its sensory threshold. Most of *DMS* in wort entered the secondary steam condensate during the evaporation process, and only a small part of *DMS* was lost due to incomplete condensation.

(2) The removal effect of *DMS* in wort is related to the film-forming effect of the multi-layer centrifugal film-forming device. An obvious correlation was observed between the *DMS* removal rate and the self-evaporation rate when the film-forming effect was good.

(3) Compared with traditional boiling, the TBA value of wort could be reduced by more than 15% without obvious oxidation using the multi-layer centrifugal film-forming device combined with holding temperature boiling.

(4) Compared with traditional boiling, the multi-layer centrifugal film-forming device could save about 216.4 kJ/L of energy consumption during the boiling and cooling process.

## Figures and Tables

**Table 1:** Test reagent required for the experiment.

Name	Specifications	Manufacturer	Purpose
DMS standard solution	≥99%	Sigma-Aldrich Chemie Gmbh (St. Louis, MO, USA)	DMS detection
Methyl ethyl sulfide	≥98%	Beijing Budweiser Technology Co., Ltd. (Beijing, China)	DMS detection
Absolute ethanol	99.5%	Shanghai McLean Biochemical Technology Co., Ltd. (Shanghai, China)	DMS detection
Sodium hydroxide standard solution	1.0 mol/L	Jiangbiao Testing Technology Co., Ltd. (China)	DMS detection
2-thiobarbituric acid	≥98%	Sigma-Aldrich Chemie Gmbh (St. Louis, MO, USA)	TBA detection
Glacial acetic acid	≥99.5%	Shanghai McLean Biochemical Technology Co., Ltd. (Shanghai, China)	TBA detection
Ultra-pure water	Self-prepared	Sichuan YOUPU Chao chun Technology Co., Ltd. (Chengdu, China)	DMS/TBA/Chromaticity detection

**Table 2:** Changes in the wort outlet temperature and energy consumption.

Speed (r/min)	Wort Outlet Temperature -TI2 (°C)		Sensible Heat Released from Wort (kJ/h)		Latent Heat of Steam (kJ/h)	
	40 L/h	80 L/h	40 L/h	80 L/h	40 L/h	80 L/h
200	86.6	90.5	1562.41	2193.63	1354.32	1925.84
400	86.1	89.7	1644.94	2460.29	1434.09	2166.91
600	85.8	88.6	1694.09	2825.25	1497.29	2531.04
800	84.9	86.4	1842.04	3551.51	1641.75	3214.25
1000	83.9	86.2	2005.44	3616.68	1820.79	3304.54
1200	83.2	86.0	2119.58	3682.09	1939.70	3379.75

**Table 3:** Comparison of the evaporation energy consumption of different boiling technologies.

Boiling System	Temperature (°C)	Boiling Time (min)	Total Evaporation Rate (%)	Evaporation Energy Consumption (kJ/L)	Remarks
Atmospheric boiling [31]	100	60–80	8	187.9	Classical
High temperature [31]	130–140	2.5–3	6–8	140.9–187.9	
low pressure boiling [31]	103–104	55–65	6–7	140.9–164.4	Classical
Low pressure [31]	104–104	45–50	4.5	105.7	Dynamic
Meura system (packing column) [8,31]	100	40–45	2	47.0	0.5~2% primary steam stripping
Tray rectification column [8,10]	100	90	2.7–4.5	63.4–105.7	Secondary steam stripping
Ziemann system [31]	100	40–50	4	93.9	Vacuum
Nerb system [31]	103/99	50–60	4.7–5.4	110.4–126.8	Vacuum
Schulz system [31]	97.5	60	1	23.5	7% vacuum evaporation
Merlin system [9,31]	100	35	5–6	117.4–140.9	Film stripping
Turbulence trickle-film stripping [15]	96	60–90	4	93.9	Gas + self-evaporation
Multilayer centrifugal film forming device	98	60	1	23.5	self-evaporation

## Data Availability

The data presented in this study are available on request from the corresponding author.
